# 17-ABAG, a novel geldanamycin derivative, inhibits LNCaP-cell proliferation through heat shock protein 90 inhibition

**DOI:** 10.3892/ijmm.2015.2239

**Published:** 2015-06-09

**Authors:** ZHIYUAN LIN, RUIXIAN PENG, ZHENYU LI, YANG WANG, CHUNHUA LU, YUEMAO SHEN, JIFENG WANG, GUOWEI SHI

**Affiliations:** 1 Department of Urology, The Fifth People’s Hospital of Shanghai, Fudan University, Shanghai 200240, P.R. China; 2 Urology Research Center, Fudan University, Shanghai 200240, P.R. China; 3 Key Laboratory of Chemical Biology, Ministry of Education, School of Pharmaceutical Sciences, Shandong University, Jinan, Shandong 250012, P.R. China

**Keywords:** geldanamycin derivative, heat shock protein 90, androgen receptor, prostate cancer, cancer treatment

## Abstract

Prostate cancer is one of the most common cancer types worldwide. In 2014, there were an estimated 233,000 new cases and 29,480 mortalities in the United States. Androgen deprivation therapy, also called androgen suppression therapy, targets androgen signaling and remains the standard treatment for patients with advanced prostate cancer; however, responses to treatment are not durable and most patients advance to castrate-resistant prostate cancer. Therefore, novel therapeutic strategies to treat prostate cancer are urgently required. Heat shock protein 90 (Hsp90) is a chaperone protein that has been shown to regulate the progression of tumor cells. Numerous Hsp90 inhibitors show anti-tumor activity and several of them have entered clinical trials. Geldanamycin (GA) was identified as the first Hsp90 inhibitor, but shows hepatotoxicity at its effective concentrations, limiting its clinical use. In previous studies by our group, the GA derivative 17-ABAG was designed and synthesized. The present study showed that 17-ABAG inhibits the proliferation and induces apoptosis of LNCaP, an androgen-dependent prostate cancer cell line, *in vitro* through a classic apoptotic pathway. 17-ABAG also downregulated the Hsp90 client protein and inhibited androgen receptor nuclear localization in LNCaP cells. In addition, 17-ABAG suppressed the growth of LNCaP xenograft tumors without any obvious side-effects. The present study demonstrated that 17-ABAG is a promising anti-tumor agent and warrants further validation in prospective studies.

## Introduction

Prostate cancer is on of the most common cancer types worldwide and is the second leading cause of cancer-associated mortalities in men in the United States (1). Hormonal therapy remains the standard therapy for patients with advanced prostate cancer by targeting androgen signaling; however, despite initial short-term clinical responses, most of the patients recur with castrate-resistant prostate cancer (2). Thus, there is a requirement to develop novel therapeutic agents to treat prostate cancer.

Heat shock protein 90 (Hsp90) is an adenosine triphosphatase (ATPase)-dependent molecular chaperone that is required for protein folding and maturation, and can interact with numerous client proteins to prevent their aggregation (3). With the client proteins, overexpression of Hsp90 is associated with the progression of tumor cells, including their survival, proliferation, invasion and metastasis (4). The androgen receptor (AR) is a nuclear receptor that has a key role in prostate cancer carcinogenesis and progression, and ARs translocate from the cytoplasm into the nucleus after activation by androgenic hormones. The AR has been shown to be a client protein of Hsp90, and Hsp90 inhibition blocks the androgen-induced nuclear import of ARs (5). Therefore, Hsp90 has been utilized as a molecular target of anti-cancer drugs and the development of an Hsp90 inhibitors has become an active area of research.

As Hsp90 is ubiquitously expressed in various cell types, selectively inhibiting cancer cell proliferation and progression by using Hsp90 inhibitors was initially considered questionable; however, it was demonstrated that tumor cells are more sensitive to Hsp90 inhibitors than normal cells (3). The reasons for therapeutic selectivity for cancer versus normal cells can be summarized as follows: Cancer cells are addicted to the oncogenic processes that are induced by oncoproteins (6). As numerous oncoproteins are Hsp90 client proteins, Hsp90 inhibition can deplete these oncoproteins and cause a greater impact on cancer cells than on normal cells (7,8). Furthermore, hypoxic, acidic and nutrient-deprived conditions are common in the tumor microenvironment and may further increase the number of denatured proteins in tumors. In order to cope with these environmental stresses, cancer cells become more dependent on Hsp90 than normal cells (9). Finally, Hsp90 inhibitors can selectively accumulate in tumor tissue while being rapidly cleared from the circulation and normal tissue (3,10), partly because Hsp90 isolated from tumor cells has a higher affinity to Hsp90 inhibitors than Hsp90 isolated from normal cells (3).

**Figure 1:**
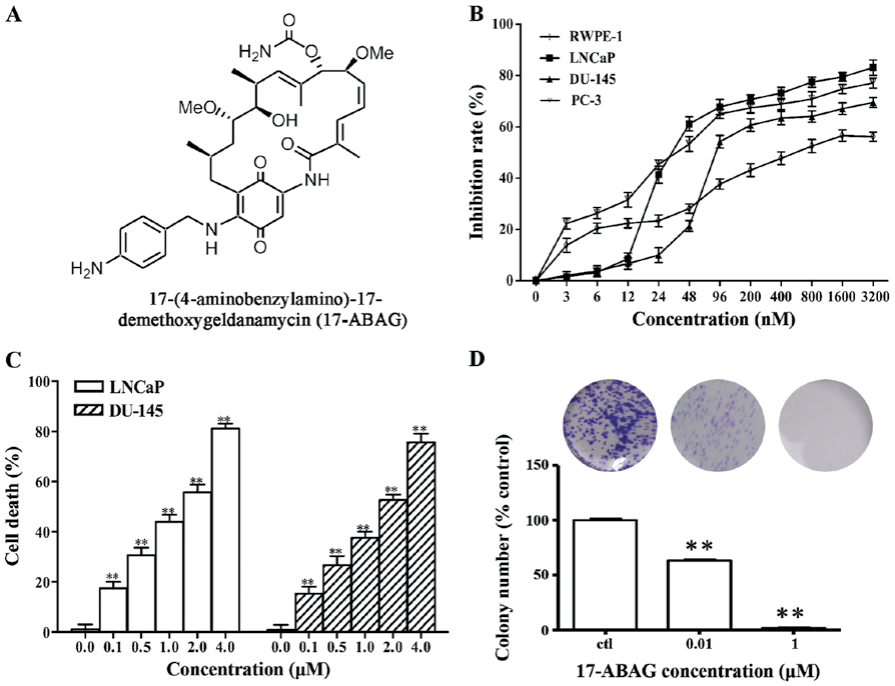
17-ABAG inhibits LNCaP cell proliferation. (A) Chemical structure of 17-ABAG. (B) The viability of RWPE-1, LNCaP, DU145 and PC-3 cells following 72-h exposure to various concentrations of 17-ABAG. The cell survival rate was assessed using the MTT assay. (C) Flow cytometric analysis of 17-ABAG-induced cell death of LNCaP and DU145 cells after treatment with various 17-ABAG concentrations for 24 h using PI staining. (D) Colony-forming capability of LNCaP cells was measured using a colony formation assay after treatment with various 17-ABAG concentrations for 10 days. Representative wells are shown in the upper panel, and quantitative analysis of the relative colony number for each group is shown in the lower panel. Values are expressed as the mean ± standard deviation (n=3). ^**^P<0.01 vs. the control.

Geldanamycin (GA) was identified as the first natural product inhibitor of Hsp90 that binds to the N-terminal ATPase domain of Hsp90 to inhibit its chaperone function, and significantly induces tumor cell death via an apoptotic mechanism (11,12). However, GA exhibits hepatotoxicity at its effective concentrations, thus limiting its clinical application (13). The modification of position 17 of GA not only leads to the retention of its the excellent anti-tumor activity but also to a reduction of its hepatotoxicity (14). According to this effect, numerous GA derivatives with reduced hepatotoxicity have been designed, and several of them have entered clinical trials to treat patients with prostate cancer (15–17). In previous studies by our group >200 GA derivatives have been designed and synthesized (14,18,19). After screening, 17-ABAG (Fig. 1A) was further examined for its *in vitro* and *in vivo* anti-cancer activities. The present study further examined the activity and mechanism of action of 17-ABAG, which showed potent anti-tumor activity against prostate cancer and low hepatotoxicity *in vivo*. Collectively, the present study provided a theoretical foundation for targeted therapies for prostate cancer and indicated that 17-ABAG is a potent, novel inhibitor of Hsp90.

## Materials and methods

17-ABAG was dissolved in dimethyl sulfoxide (DMSO; Sigma-Aldrich, St. Louis, MO, USA) to prepare 10-mmol/l stock solutions that were stored at −20°C.

### Details of antibodies and reagents

Anti-β-actin antibody (sc-47778, monoclonal, raised in mouse, 1:10,000) was from Santa Cruz Biotechnology, Inc. (Dallas, TX, USA), MTT, Hoechst 33258 and RNaseA were all purchased from Sigma-Aldrich. Anti-AKT (#9272, polyclonal, raised in rabbit, 1:1,000), anti-phospho (p)-AKT (ser473; #4058, monoclonal, raised in rabbit, 1:1,000), anti-human epidermal growth factor receptor 2 (Her2; #2165, monoclonal, raised in rabbit, 1:1,000), anti-epidermal growth factor receptor (EGFR; sc-03, polyclonal, raised in rabbit, 1:500), anti-c-Raf (#9422, polyclonal, raised in rabbit, 1:1,000), anti-B-cell lymphoma 2 (Bcl-2; #2876, polyclonal, raised in rabbit, 1:1,000) and anti-Bcl-2- associated X protein (Bax; #2772, polyclonal, raised in rabbit, 1:1,000) antibodies were from Cell Signaling Technology (Beverly, MA, USA). Anti-cyclin-dependent kinase 4 (Cdk4; sc-260, polyclonal, raised in rabbit, 1:500), and anti-prostate-specific antigen (PSA; sc-7316, monoclonal, raised in mouse, 1:500), anti-AR (sc-7305, monoclonal, raised in mouse, 1:500), anti-Hsp70 (sc-24; monoclonal, raised in mouse, 1:500), Hsp90 (sc-69703, monoclonal, raised in mouse, 1:500) and NKX-3.1 (sc-15022, polyclonal, raised in goat, 1:500) antibodies were purchased from Santa Cruz Biotechnology, Inc. The Annexin V fluorescein isothiocyanate (FITC) Apoptosis Detection kit was purchased from BD Pharmingen (San Diego, CA, USA).

### Cells and cell culture

The human androgen-dependent prostate cancer cell line, LNCaP, two human androgen-independent prostate cancer cell lines, DU145 and PC-3, and the normal human prostate cell line, RWPE-1, were purchased from the American Type Culture Collection (Manassas, VA, USA). The cells were all maintained in Roswell Park Memorial Institute (RPMI)-1640 medium (Gibco/Invitrogen, Mount Waverley VIC, Australia). The medium was supplemented with 10% fetal bovine serum (FBS, Gibco) 2 mM L-glutamine (Gibco), penicillin (100 units/ml) and streptomycin (100 *μ*g/ml, Biowest LLC, Kansas City, MO, USA). The cells were incubated at 37°C in an atmosphere of 5% CO_2_ and 95% air.

### Cytotoxicity assays

The cytotoxicity of the compounds was measured using an MTT assay (Sigma-Aldrich) as previously described (20). DNA content was detected using a FCM-FC500 system (Beckman Coulter, Brea, CA, USA)

### Measurement of cell death

Cell death induced by compounds was determined by evaluating the plasma membrane integrity by examining the permeability of cells to propidium iodide (PI). Cells were trypsinized, collected and centrifuged at 300 × g at 4°C for 5 min, washed once with phosphate-buffered saline (PBS) and re-suspended in PBS containing 5 *μ*g/ml PI. The level of PI incorporation was quantified by flow cytometry using a FACScan flow cytometer (Beckman Coulter EPICS XL; Beckman Coulter).

### Colony formation assay

Cells were cultured in six-well plates (1,000/well) overnight, followed by replacement of the medium with added 17-ABAG. The plates were then incubated at 37°C with 5% CO_2_ for 10 days. On the last day, the medium was removed, and after washing with PBS and fixing with methanol, the colonies were stained with crystal violet solution (Sangon Biotech, Shanghai, China) for 3 h at room temperature. The cells were observed under a microscope (Leica DMIL; Leica Microsystems, Wetzlar, Germany) and images were captured with a scanner (Leica Application Suite version 4.40; Leica Microsystems) and visible colony numbers were counted after washing and air-drying.

### Detection of apoptosis by DAPI staining

Following treatment with 17-ABAG, the cells were collected and washed once with 2 ml of ice-cold PBS, fixed with 1 ml 4% paraformaldehyde for 20 min and washed once again with 2 ml ice-cold PBS. The cells were incubated in 1 ml PBS containing DAPI at 50 *μ*g/ml and 100 *μ*g/ml RNase A (both from Sigma-Aldrich). This mixture was incubated for 30 min at 37°C. After washing with 2 ml PBS three times, the cells were observed using a fluorescence microscope (Leica DM2500; Leica Microsystems) at 340 nm (excitation) and 488 nm (emission).

### Assessment of apoptosis by Annexin V/PI staining

Cells were seeded in a 6-well plate 1 day prior treatment with the compounds. After 17-ABAG treatment for 24 h, cells were stained with Annexin V and PI following the manufacturer’s instructions (Annexin V-FITC Apoptosis Detection kit; BD Pharmingen). Subsequently, cells were analyzed by flow cytometry and BD CellQuest Pro software (BD Pharmingen) using the FL1 channel for FITC and FL3 detector for PI.

### Western blot analysis

After treatment with or without 17-ABAG for different durations, cells were harvested and lysed in ice-cold lysis buffer [20 mM Tris-HCl (pH 7.4), 150 mM NaCl, 1 mM EDTA, 1 mM ethylene glycol tetraacetic acid, 1% Triton X-100, 2.5 mM sodium pyrophosphate, 1 mM β-glycerolphosphate, 1 mM sodium orthovanadate, 1 mg/ml leupeptin and 1 mM phenylmethylsulfonyl fluoride; Beyotime Biotechnology, Shanghai, China]. The lysate was mixed with an equal volume of 2X loading buffer [4% SDS, 10% 2-mercaptoethanol, 20% glycerol and 0.2 mg/ml bromophenol blue in 0.1 M Tris-HCl (pH 6.8)] and boiled for 10 min immediately. The boiled lysates were separated by 8–12% SDS-PAGE at 100 V and then were transferred onto Immobilon-P membranes (Millipore, Billerica, MA, USA). After blocking the membranes with 5% skimmed milk in PBS with 0.1% Tween-20 for 1 h, they were incubated overnight with the corresponding primary antibodies in blocking solution at 4°C. Antibodies against the following proteins were obtained from Santa Cruz Biotechnology, Inc.: EGFR, Hsp70, AR, Hsp90, PSA, NKX-3.1 and Cdk4. Antibodies against the following proteins were from Cell Signaling Technology (Danvers, MA): Bax, Bcl-2, HER2, phospho-Akt, Akt and c-Raf. The primary antibodies were detected using either a peroxidase-conjugated ImmunoPure goat anti-rabbit immunoglobulin G (IgG) (H+L) or peroxidase-conjugated ImmunoPure goat anti-mouse IgG (H+L) secondary antibody and enhanced chemiluminescence (Western ECL reagent, WBKL0500, Millipore). TBST was used for washing between the addition of the primary and secondary antibodies. The Fluor Chem-E western imaging system (ProteinSimple, Santa Clara, CA) was used to capture images.

### Reverse transcription quantitative polymerase chain reaction (PCR)

Total RNA from 2×10^6^ cells for each cell line was isolated using TRIzol reagent (Invitrogen Life Technologies, Carlsbad, CA, USA). Two micrograms of total RNA were reverse transcribed using the Transcriptor First Strand cDNA synthesis kit (Roche Applied Science, Basel, Switzerland). To synthesize thecDNA, 0.5 mM deoxynucleoside triphosphate, 50 pmol random hexamers, 50 U ExScript reverse transcriptase (200 U/μl), 10 U RNase inhibitor, 500 ng total RNA, and 1X reaction buffer were mixed in each reaction tube (10 *μ*l per reaction) and then incubated at 42°C for 15 min, followed by a 2-min incubation at 95°C to inactivate the ExScript reverse transcriptase. Real-time monitoring of PCR amplification of the cDNA was performed using DNA primers and the ABI PRISM 7500 HT Sequence Detection system (Applied Biosystems; Foster City, CA, USA) with SYBR PCR Master Mix (Thermo Fisher Scientific, Waltham, MA, USA) using the following program: 95°C for 10 sec, 1 cycle; 95°C for 5 sec, 62°C for 31 sec, 40 cycles; followed by a 30-min melting curve collection to verify the primer dimers. Target gene expression was normalized to GAPDH levels in the respective samples as an internal standard, and the comparative cycle threshold (Ct) method was used to calculate relative amount of target mRNAs, as previously described (21). Oligonu cleotide primers used for PCR amplification of human GAPDH, PSA, NKX3.1 and FKBP5 were as follows: GAPDH sense, 5′-TCCTGTTCGACAGTCAGCCGCA-3′ and antisense, 5′-ACCAGGCGCCCAATACGACCA-3′; PSA sense, 5′-CACAGCCTGTTTCATCCTGA-3′ and antisense, 5′-AGGTCCATGACCTTCACAGC-3′; NKX3.1 sense, 5′-GGACTGAGTGAGCCTTTTGC-3′ and antisense, 5′-CAGCCAGATTTCTCCTTTGC-3′; FKBP5 sense, 5′-TCCCTCGAATGCAACTCTCT-3′ and antisense, 5′-GCCACATCTCTGCAGTCAAA-3′. Each PCR reaction was carried out in triplicate.

### Immunofluorescence

LNCaP cells were grown on coverslips and treated with or without 0.2 *μ*M 17-ABAG for 24 h followed by treatment with 1 nmol/l R1881 for 6 h. After treatment, cells were fixed with 4% formaldehyde, permeabilized for 10 min in 0.2% Triton X-100 in PBS and then incubated for 1 h in blocking buffer (5% bovine serum albumin in PBS). Next, the cells were incubated with AR (1:250) antibody overnight, and were then visualized with Cy3-conjugated addinipure goat anti-mouse IgG (H+L). Nuclei were stained by incubating the cells with 10 *μ*g/ml Hoechst 33258 (Sigma-Aldrich) in PBS and then washing extensively with PBS. Images were captured using a fluorescence microscope (Leica DM2500; Leica Microsystems).

### In vivo anti-tumor assays

Six- to eight-week-old male athymic nude mice (BALB/c-nu; n=14) were obtained from Slac Laboratory Animal (Shanghai, China). The mice were kept in cages (97 cm^2^×12.7 cm) in an environment with a temperature of 26–28°C, a humidity of 40–60% and a 10 hlight/14 h dark cycle and were allowed free access to food and water (every other day). LNCaP or DU-145 cells (1×10^6^ cells/animal) were injected subcutaneously to generate orthotopic xenografts. Next, the mice bearing tumor cells were randomly divided into treatment and control groups (7 mice per group). The drug was injected via the caudal vein every three days at a dose of 10 mg/kg body weight, whereas the blank control group received an equal volume of 5% glucose (Tianjin Pacific Pharmaceutical, Tianjin, China) injection containing 1% DMSO and 2% lecithin (Sangon Biotech). During treatment, subcutaneous tumors were measured with a vernier caliper every three days, and body weight was monitored regularly (the mean weight on day 0 was 25.287 (control) vs. 24.646 (treated group), and on day 21 it was 25.621 (control) vs. 25.468 (treated group). The tumor volume was calculated by the formula (V=ab^2^/2, where a and b represent the longest and shortest diameters of the tumor, respectively). After treatment for 21 days with the drug, the animals were sacrificed by spinal dislocation and solid tumors were removed. All of the animal protocols of the present study were approved by the Shanghai Medical Experimental Animal Care Commission (Shanghai, China).

### Immunohistochemical staining

Sections of the heart, liver, spleen, lung, kidney and tumor were prepared for immunohistochemical analysis from sacrificed mice with tumor xenografts as previously described (22). The expression of Ki67 (Santa Cruz Inc, sc-15402) were detected by immunohistochemical staining. The paraffin-embedded sections were pre-treated and stained with antibodies. The secondary antibodies against rabbit IgG were supplied in an IHC kit (#CW2069) from Beijing Cowin Bioscience Co., Ltd., Beijing, China, and the sections were observed using a Leica DM2500 microscope (Leica Microsystems).

### Statistical analysis

Values are expressed as the means ± standard error of the mean. Student’s t-test (SPSS 19.0, IBM) was used to determine the significant differences between the treatment and control groups. P<0.05 was considered to indicate a statistically significant difference between values. All of the experiments were conducted in triplicate.

## Results

### 17-ABAG inhibits LNCaP cell proliferation

To evaluate the *in vitro* anti-tumor effects of 17-ABAG, MTT assays were performed to examine the proliferative inhibitory activity of 17-ABAG against the normal human prostate cell line RWPE-1 and the three human prostate cancer cell lines LNCaP, DU-145 and PC-3. In all of the cell lines tested, 17-ABAG inhibited the cell growth in a dose-dependent manner. In the prostate cancer cell lines, 17-ABAG displayed potent cytotoxicity with half maximal inhibitory concentration (IC_50_) values ranging from 30.15 to 102.63 nmol/l (LNCaP, 30.15 nM; DU-145, 102.63 nM; PC-3, 44.27 nM) at 72 h (Fig. 1B). However, 17-ABAG showed lower cytotoxicity to RWPE-1 cells, with an IC_50_ value of 589 nM (Fig. 1B). These results indicated that 17-ABAG possesses high activity against LNCaP cells but lower cytotoxicity against normal prostate cells (RWPE-1).

To assess the ability of 17-ABAG to induce cell death, membrane integrity was assessed using PI staining. The results showed that 17-ABAG induced cell death of LNCaP and DU-145 cells in a dose-dependent manner (Fig. 1C), suggesting that cell death is the main contributor to the anti-proliferative activity of 17-ABAG. Consistently, a colony formation assay showed that the numbers of colonies formed by the cells treated with 17-ABAG significantly decreased compared with that of the control LNCaP (Fig. 1D) and Du-145 cells (data not shown).

### 17-ABAG induces LNCaP cell apoptosis

**Figure 2:**
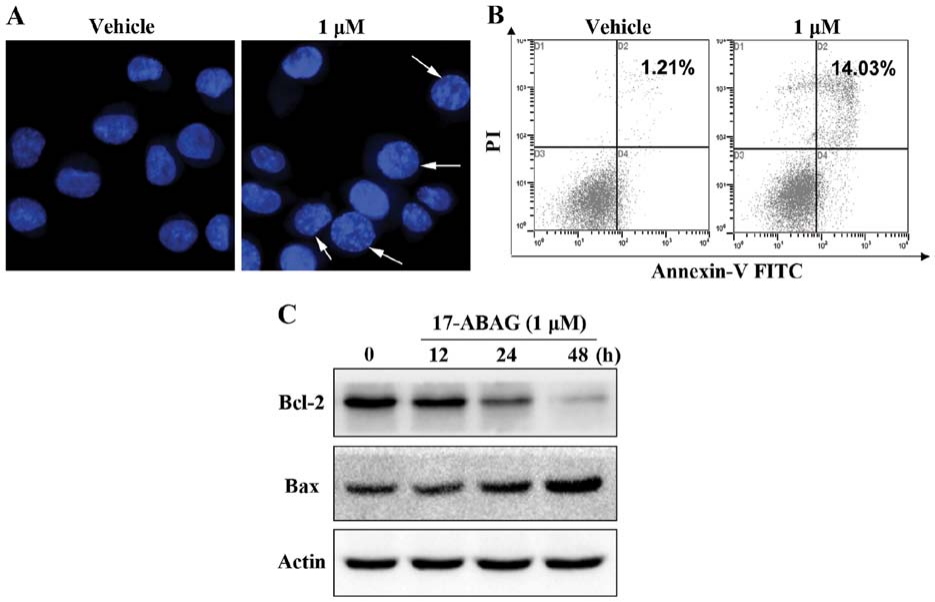
17-ABAG induces prostate cancer cell apoptosis. (A) LNCaP cells were treated for 24 h with or without 1 *μ*M 17-ABAG. The images were captured using fluorescence microscopy (magnification, ×600) to observe nuclear staining with DAPI. Apoptotic cells with condensed nuclei were identified using nuclear staining as indicated by the white arrows. (B) Detection of apoptosis using Annexin V-FITC/PI double staining. After treatment with or without 1 *μ*M 17-ABAG for 24 h, LNCaP cells were analyzed using a FACSCalibur flow cytometer. The horizontal axis represents Annexin V intensity, and the vertical axis shows PI staining. The lines divide each plot into four quadrants: Lower left quadrant, live cells; lower right quadrant, early apoptotic cells; upper left quadrant, necrotic cells; upper right quadrant, late apoptotic cells. (C) Western blot analysis of apoptosis-associated proteins after treatment with 17-ABAG. DU-145 cells were treated for the indicated times with 1 *μ*M 17-ABAG. The DU-145 cells were treated for the indicated periods of time with 1 *μ*M 17-ABAG. The expression levels of Bcl-2 and Bax were determined by western blot analysis. β-actin was used for normalization and verification of protein loading. PI, propidium iodide; Bcl-2, B-cell lymphoma 2; Bax, Bcl-2-associated X protein; FITC, fluorescein isothiocyanate.

Induction of apoptosis is one of the important mechanisms via which chemotherapeutic drugs kill tumor cells. DAPI staining revealed that 17-ABAG induced morphological changes in the cells within 24 h of exposure (Fig. 2A). The cells shrank, became rounded and contained fragmented nuclei, all of which are characteristic morphological features (i.e., condensed nuclei) of stressed cells moving into apoptosis. These observations led to the hypothesis that 17-ABAG induces apoptosis of LNCaP cells. Flow cytometry and western blot analyses were utilized to explore whether the anti-proliferative activity of 17-ABAG is associated with apoptosis. The Annexin V-PI assay revealed that the number of cells undergoing apoptosis significantly increased following treatment with 17-ABAG compared with that in the control LNCaP cells (Fig. 2B). In addition, 17-ABAG increased the expression levels of apoptosis-associated protein Bcl-2 and reduced the expression levels of Bax (Fig. 2C). These results suggested that 17-ABAG induces apoptosis via the classic apoptotic pathway.

### 17-ABAG downregulates Hsp90 client proteins

**Figure 3:**
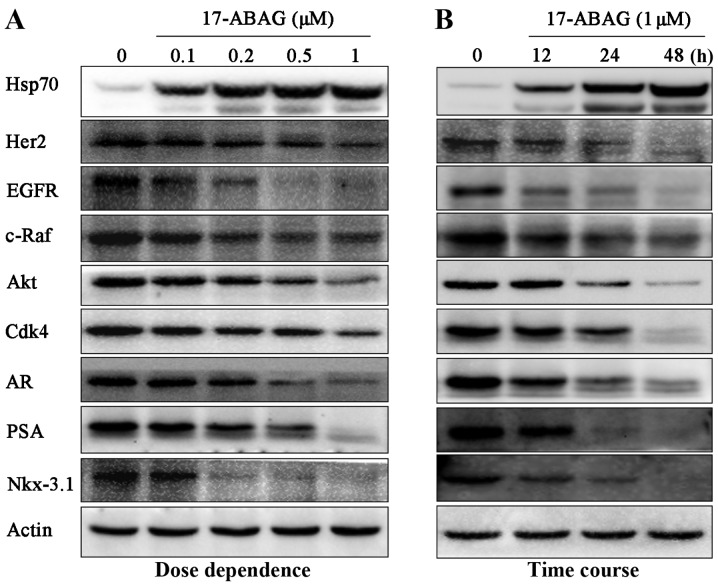
17-ABAG induces the degradation of Hsp90 client proteins. (A) LNCaP cells were cultured in the presence of 17-ABAG at the indicated concentrations for 24 h, and the proteins regulated by Hsp90 or ARs were assessed by western blot analysis. β-actin was used for normalization and verification of protein loading. (B) LNCaP cells were treated with 1 *μ*M 17-ABAG for the indicated times and the proteins regulated by Hsp90 or ARs were assessed by western blot analysis. β-actin was used for normalization and verification of protein loading. HSP, heat shock protein; AR, androgen receptor; PSA, prostate-specific antigen; EGFR, epidermal growth factor receptor; Cdk4, cyclin-dependent kinase 4; Her2, human epidermal growth factor receptor 2; Nkx-3.1, Nk3 homeobox 1.

GA has been identified as an Hsp90 inhibitor via facilitating the degradation of Hsp90 client proteins required for tumor growth (11,12). To determine whether 17-ABAG can also regulate Hsp90, the client proteins of Hsp90 were examined, including Her2, EGFR, c-Raf, AKT, p-AKT and Cdk4. Western blot analysis demonstrated that these well-known client proteins were significantly downregulated following treatment with 17-ABAG in a time- and dose-dependent manner (Fig. 3), demonstrating that 17-ABAG induces Hsp90 client protein degradation.

### 17-ABAG inhibits AR signaling in LNCaP cells

**Figure 4:**
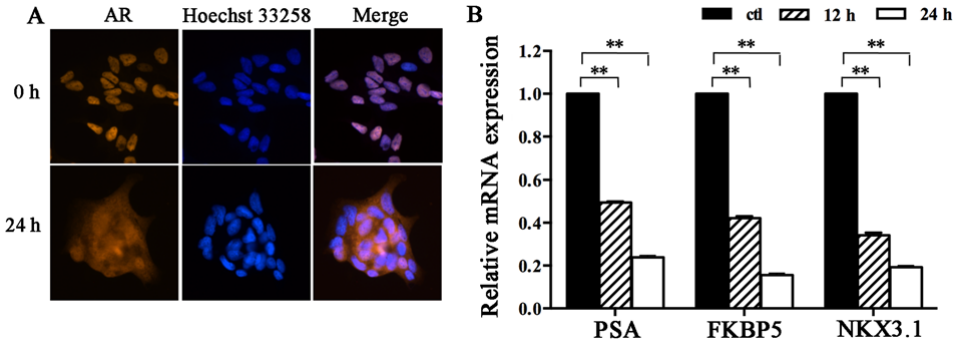
17-ABAG inhibits the AR pathway in LNCaP cells. (A) LNCaP cells were treated with 0.2 *μ*M 17-ABAG for 0 or 24 h, followed by treatment with 1 nM R1881, a potent non-aromatizable androgen, for 6 h. The nuclei were stained with Hoechst 33258 and AR localization was assessed using immunofluorescence (magnification, ×400). (B) LNCaP cells were treated with 1 *μ*M 17-ABAG for 12 or 24 h, and PSA, FKBP5 and NKX3.1 mRNA levels were determined using quantitative real-time polymerase chain reaction. The mRNA levels were normalized to GAPDH mRNA. Values are expressed as the mean ± standard error of the mean. All of the experiments were repeated at least three times. ^**^P<0.01 vs. Ctl. Ctl, control; AR, androgen receptor.

AR has been reported to be a client protein of Hsp90 (5). Therefore, the present study next evaluated the effects of 17-ABAG on AR signaling in LNCaP cells. 17-ABAG was shown to induce AR downregulation in LNCaP cells (Fig. 3). Furthermore, immunofluorescence staining indicated that AR nuclear translocation was blocked after treatment with 17-ABAG. Abundant AR residues were observed in the nucleus following staining with R1881, a photoaffinity label of AR, while the nuclear translocation of ARs was significantly inhibited by pre-treatment with 17-ABAG (Fig. 4A). This observation indicated that 17-ABAG inhibited the nuclear localization of AR in LNCaP cells *in vitro*, which was consistent with the results of a previous study (5).

To further confirm the effect of 17-ABAG on AR function, the mRNA levels of several well-characterized androgen-regulated genes were measured in LNCaP cells. RT-qPCR indicated that 17-ABAG significantly decreased the mRNA levels of AR target genes, including PSA, NKX3.1 and FKBP5 (Fig. 4B), suggesting that AR transcriptional activity was blocked by 17-ABAG treatment. The protein levels of the AR target genes PSA and NKX3.1 were also examined. Western blot analysis demonstrated that the expression levels of these AR target genes were significantly downregulated following treatment with 17-ABAG in a time- and dose-dependent manner (Fig. 3).

### 17-ABAG is a promising anti-tumor agent in vivo

Based on the potent inhibitory *in vitro* effects of 17-ABAG on the LNCaP cell line, the *in vivo* anti-tumor activity of 17-ABAG was evaluated using prostate cancer xenografts of LNCaP cells.

**Figure 5:**
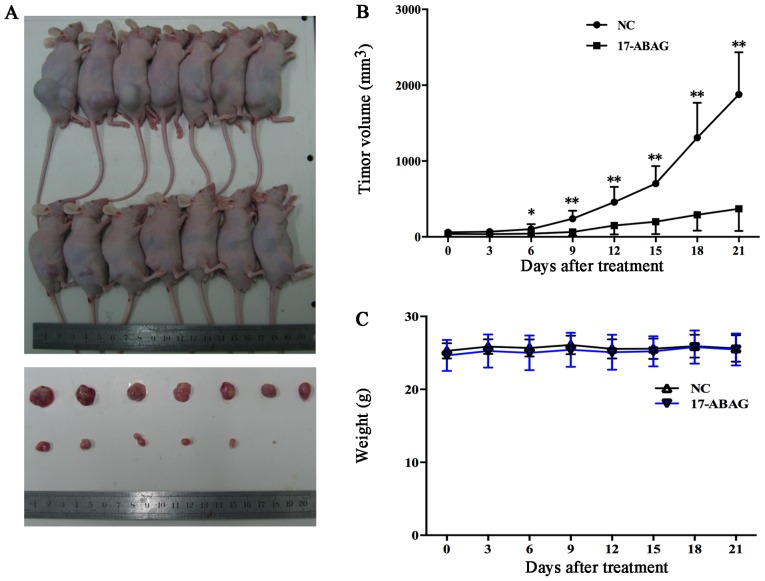

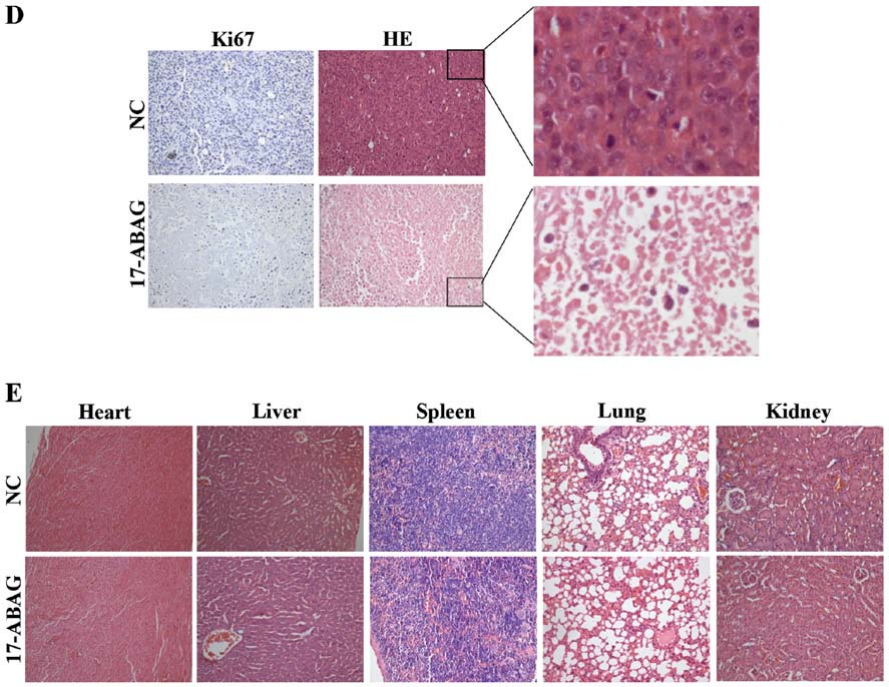
17-ABAG significantly delays LNCaP tumor growth. (A) 17-ABAG inhibits the growth of LNCaP xenograft tumors in mice. Upper panel, images of tumor-bearing mice with (bottom row) or without (top row) 17-ABAG treatment. Lower panel, images of extracted LNCaP tumors with (bottom row) or without (top row) 17-ABAG treatment. (B) Tumor volume of the mice. The animals treated with 17-ABAG exhibited a significant decrease in average tumor volume compared with that in the control mice from day six onwards. ^*^P<0.05. (C) Body weight of the mice. P>0.05 (17-ABAG treated vs. control). Values are expressed as the mean ± standard error of the mean (n=7). (D) HE staining and anti-Ki67 immunohistological staining analysis of tumor sections from the mice (magnification, ×200). (E) HE-stained sections of the heart, liver, spleen, lung and kidney of the mice (magnification, ×200). HE, hematoxylin and eosin; NC, negative control.

LNCaP cells were sub-cutaneously inoculated into male nude mice, which received an injection of either vehicle control or 17-ABAG (10 mg/kg every three days). The animals treated with 17-ABAG (n=7) exhibited a significantly lower average tumor volume compared with the control mice from day six onwards (42.68 vs. 102.08 mm^3^, respectively; P<0.05) (Fig. 5A). After 21 days’ treatment, the average tumor volume was 370.09 mm^3^ for treated mice compared with 1,876.87 mm^3^ for control mice (Fig. 5A and B). When each animal was considered individually, the incidence of mice progressing with a tumor volume of 900 mm^3^ or greater was significantly diminished by day 21 in 17-ABAG-treated animals (0/7; 0%) compared with controls (7/7; 100%). No obvious side-effects or body weight loss were observed (Fig. 5C). Immunohistochemical analysis indicated decreased Ki67 expression after treatment with 17-ABAG in the tumors *in vivo* (Fig. 5D). The observed inhibition of tumor progression by 17-ABAG may have resulted from decreased proliferation (reduced Ki67, the proliferation marker). More importantly, no damage to the organs was detected, including the heart, liver, spleen, lung and kidney (Fig. 5E). These results showed that 17-ABAG was successful in suppressing the growth of LNCaP xenograft tumors. These findings suggested that 17-ABAG suppressed prostate tumor growth *in vivo* without any observable side-effects.

## Discussion

Prostate cancer is one of the most common cancer types in males worldwide, occurring more commonly in the developed world and at increasing rates in developing countries (23,24). Beginning with Huggins and Hodges (25) first reporting the susceptibility of prostate cancer to androgen withdrawal, hormonal therapy remains the most effective therapy for patients with advanced prostate cancer. However, after 12–18 months on average, a large percentage of prostate cancer patients will eventually progress to a castration-resistant stage and succumb to the disease (median survival time, ~1–2 years) (2,26). There are limited therapeutic options available for castrate-resistant prostate cancer (CRPC). Chemotherapy drugs, immunotherapy and vaccine therapy exhibit limited efficacy and limited improvement in survival (27). Thus, novel approaches for the treatment of patients with advanced disease are still urgently required. With a deeper understanding of the molecular mechanisms of the tumorigenesis and progression of prostate cancer, numerous approaches, preferably based on selective targeting of mechanistically relevant cancer proteins, are currently being evaluated to improve the treatment of prostate cancer (28).

Hsp90 is a molecular chaperone that maintains the normal activity of cells through ensuring the proper folding, maturation, conformational stabilization and location of its client proteins (3,29). However, through a variety of oncoproteins, Hsp90 can regulate the progression of tumor cells and affect their survival, proliferation, invasion and metastasis (4,29–31). Previous studies have shown that in prostate cancer, Hsp90 regulates the nuclear localization and activation of ARs (5,32,33), which has a key role in prostate carcinogenesis and progression (34,35). The mechanisms of Hsp90 action in prostate cancer are likely to be complex, as AKT and Her-2 signaling are also important pathways in prostate cancer and, of note, are Hsp90 client proteins (5,36).

Cancer has six major hallmarks (37), and Hsp90 inhibitors appear to be the only cancer chemotherapeutic agents known to produce strong combinatorial effects on all of the hallmarks of cancer simultaneously (38). In addition, the multiple downstream effects markedly reduce the opportunities for cancer cells to develop resistance to Hsp90 inhibitors (39,40). Although Hsp90 is widely expressed in various cell types, the inhibition of Hsp90 can selectively kill cancer cells with little effect on normal cells (3,41). Thus, Hsp90 has been considered as a novel molecular target in prostate cancer (5,42).

GA was the first reported natural product inhibitor of Hsp90; however, it exhibits hepatotoxicity at its effective concentrations, thus limiting its clinical use. A number of Hsp90 inhibitors have demonstrated significant anti-tumor effects in multiple cancer models, many of which have been evaluated in clinical trials (15–17). However, studies have suggested that most of these inhibitors are not efficacious in monotherapy and have certain disadvantages (15–17,43–45). For example, IPI-504, a derivative of geldanamycin, showed a minimal effect on the PSA level or tumor burden and was associated with unacceptable toxicity (17). In previous studies by our group, >200 GA derivatives were designed and synthesized (14,18,19), among which 17-ABAG inhibited the proliferation and induced apoptosis of LNCaP cells *in vitro* as well as *in vivo*.

The present study demonstrated that 17-ABAG can selectively inhibit androgen-dependent (LNCaP) and androgen-independent (DU-145 and PC-3) prostate cancer cells with markedly lower cytotoxicity against normal prostate cells (RWPE-1) *in vitro*. In addition, the present study showed that 17-ABAG was able to induce LNCaP cell apoptosis through the regulation of apoptosis-associated proteins. As an Hsp90 inhibitor, 17-ABAG, also downregulated the protein levels of Hsp90 client proteins, including Her2, EGFR, AKT, c-Raf and Cdk4 in a time- and dose-dependent manner, which may partly account for the mechanism of the anti-proliferative activity of 17-ABAG against androgen-dependent prostate cancer cells.

Previous studies have demonstrated that in prostate cancer, Hsp90 regulates the nuclear localization and activation of ARs (5,32,33), which have a key role in prostate carcinogenesis and progression (34,35). The present study demonstrated that the localization of ARs shifted from predominantly nuclear to cytoplasmic after treatment with 17-ABAG, consistent with the results of previous studies. 17-ABAG also decreased the protein levels of ARs and decreased the mRNA and protein levels of AR target genes, including PSA, NKX3.1 and FKBP5.

To determine whether 17-ABAG shows any anti-tumor effects *in vivo*, LNCaP cells were subcutaneously inoculated into male nude mice, and tumor-bearing animals were treated via vein injection with 17-ABAG after the development of visible tumors. It was observed that 17-ABAG showed significant anti-tumor effects compared with the vehicle control treatment. In addition, no obvious side effects or organ damage were detected, suggesting that 17-ABAG treatment may be a potential safe treatment for prostate cancer.

In conclusion, 17-ABAG inhibited the proliferation of LNCaP cells *in vitro* and *in vivo* by decreasing the expression of Hsp90 client proteins, inhibiting the AR signaling pathway and inducing apoptosis. The results of the present study suggested that 17-ABAG may be a potential safe treatment for prostate cancer; however, its clinical potential requires validation in prospective studies.

## References

[1] Siegel R, Ma J, Zou Z, Jemal A (2014). Cancer statistics, 2014. CA Cancer J Clin.

[2] Moul JW, Dawson N (2012). Quality of life associated with treatment of castration-resistant prostate cancer: A review of the literature. Cancer Invest.

[3] Kamal A, Thao L, Sensintaffar J, Zhang L, Boehm MF, Fritz LC, Burrows FJ (2003). A high-affinity conformation of Hsp90 confers tumour selectivity on Hsp90 inhibitors. Nature.

[4] Gorska M, Popowska U, Sielicka-Dudzin A, Kuban-Jankowska A, Sawczuk W, Knap N, Cicero G, Wozniak F (2012). Geldanamycin and its derivatives as Hsp90 inhibitors. Front Biosci (Landmark Ed).

[5] Ischia J, Saad F, Gleave M (2013). The promise of heat shock protein inhibitors in the treatment of castration resistant prostate cancer. Curr Opin Urol.

[6] Weinstein IB, Joe AK (2006). Mechanisms of disease: Oncogene addiction - a rationale for molecular targeting in cancer therapy. Nat Clin Pract Oncol.

[7] da Rocha Dias S, Friedlos F, Light Y, Springer C, Workman P, Marais R (2005). Activated B-RAF is an Hsp90 client protein that is targeted by the anticancer drug 17-allylamino-17-demethoxygel-danamycin. Cancer Res.

[8] Grbovic OM, Basso AD, Sawai A, Ye Q, Friedlander P, Solit D, Rosen N (2006). V600E B-Raf requires the Hsp90 chaperone for stability and is degraded in response to Hsp90 inhibitors. Proc Natl Acad Sci USA.

[9] Whitesell L, Bagatell R, Falsey R (2003). The stress response: Implications for the clinical development of hsp90 inhibitors. Curr Cancer Drug Targets.

[10] Chiosis G, Neckers L (2006). Tumor selectivity of Hsp90 inhibitors: The explanation remains elusive. ACS Chem Biol.

[11] Scheibel T, Buchner J (1998). The Hsp90 complex - a super-chaperone machine as a novel drug target. Biochem Pharmacol.

[12] Stebbins CE, Russo AA, Schneider C, Rosen N, Hartl FU, Pavletich NP (1997). Crystal structure of an Hsp90-geldanamycin complex: Targeting of a protein chaperone by an antitumor agent. Cell.

[13] Fukuyo Y, Hunt CR, Horikoshi N (2010). Geldanamycin and its anti-cancer activities. Cancer Lett.

[14] Li Z, Jia L, Wang J, Wu X, Hao H, Xu H, Wu Y, Shi G, Lu C, Shen Y (2014). Design, synthesis and biological evaluation of 17-arylmethylamine-17-demethoxygeldanamycin derivatives as potent Hsp90 inhibitors. Eur J Med Chem.

[15] Heath EI, Hillman DW, Vaishampayan U, Sheng S, Sarkar F, Harper F, Gaskins M, Pitot HC, Tan W, Ivy SP (2008). A phase II trial of 17-allylamino-17-demethoxygeldanamycin in patients with hormone-refractory metastatic prostate cancer. Clin Cancer Res.

[16] Pacey S, Wilson RH, Walton M, Eatock MM, Hardcastle A, Zetterlund A, Arkenau HT, Moreno-Farre J, Banerji U, Roels B (2011). A phase I study of the heat shock protein 90 inhibitor alvespimycin (17-DMAG) given intravenously to patients with advanced solid tumors. Clin Cancer Res.

[17] Oh WK, Galsky MD, Stadler WM, Srinivas S, Chu F, Bubley G, Goddard J, Dunbar J, Ross RW (2011). Multicenter phase II trial of the heat shock protein 90 inhibitor, retaspimycin hydrochloride (IPI-504), in patients with castration-resistant prostate cancer. Urology.

[18] Li Z, Jia L, Wang J, Wu X, Shi G, Lu C, Shen Y (2015). Discovery of novel 17-phenylethyla minegeldanamycin derivatives as potent Hsp90 inhibitors. Chem Biol Drug Des.

[19] Wu Y, Li Z, Wang Z, Xu H, Wu X, Lu C, Shen Y (2014). Synthesis of novel 17-[3,6-Dioxa-8-N-(substituted cinnamyol)- octanediamino]-17-demethoxygeldanamycin derivatives. Chin J Org Chem.

[20] Mosmann T (1983). Rapid colorimetric assay for cellular growth and survival: Application to proliferation and cytotoxicity assays. J Immunol Methods.

[21] Mahdavinezhad A, Mousavi-Bahar SH, Poorolajal J, Yadegarazari R, Jafari M, Shabab N, Saidijam M (2015). Evaluation of miR-141, miR-200c, miR-30b Expression and Clinicopathological Features of Bladder Cancer. Int J Mol Cell Med.

[22] Shen P, Sun J, Xu G, Zhang L, Yang Z, Xia S, Wang Y, Liu Y, Shi G (2014). KLF9, a transcription factor induced in flutamide-caused cell apoptosis, inhibits AKT activation and suppresses tumor growth of prostate cancer cells. Prostate.

[23] Baade PD, Youlden DR, Krnjacki LJ (2009). International epidemiology of prostate cancer: Geographical distribution and secular trends. Mol Nutr Food Res.

[24] Jemal A, Bray F, Center MM, Ferlay J, Ward E, Forman D (2011). Global cancer statistics. CA Cancer J Clin.

[25] Huggins C, Hodges CV (1972). Studies on prostatic cancer. I. The effect of castration, of estrogen and androgen injection on serum phosphatases in metastatic carcinoma of the prostate. CA Cancer J Clin.

[26] Asmane I, Céraline J, Duclos B, Rob L, Litique V, Barthélémy P, Bergerat JP, Dufour P, Kurtz JE (2011). New strategies for medical management of castration-resistant prostate cancer. Oncology.

[27] Lassi K, Dawson NA (2010). Update on castrate-resistant prostate cancer: 2010. Curr Opin Oncol.

[28] Corcoran NM, Gleave ME (2012). Targeted therapy in prostate cancer. Histopathology.

[29] Pearl LH, Prodromou C (2006). Structure and mechanism of the Hsp90 molecular chaperone machinery. Annu Rev Biochem.

[30] Whitesell L, Lindquist SL (2005). HSP90 and the chaperoning of cancer. Nat Rev Cancer.

[31] Takayama S, Reed JC, Homma S (2003). Heat-shock proteins as regulators of apoptosis. Oncogene.

[32] Lamoureux F, Thomas C, Yin MJ, Kuruma H, Fazli L, Gleave ME, Zoubeidi A (2011). A novel HSP90 inhibitor delays castrate-resistant prostate cancer without altering serum PSA levels and inhibits osteoclastogenesis. Clin Cancer Res.

[33] Saporita AJ, Ai J, Wang Z (2007). The Hsp90 inhibitor, 17-AAG, prevents the ligand-independent nuclear localization of androgen receptor in refractory prostate cancer cells. Prostate.

[34] Cano LQ, Lavery DN, Bevan CL (2013). Mini-review: Foldosome regulation of androgen receptor action in prostate cancer. Mol Cell Endocrinol.

[35] Huang CK, Luo J, Lee SO, Chang C (2014). Concise review: Androgen receptor differential roles in stem/progenitor cells including prostate, embryonic, stromal, and hematopoietic lineages. Stem Cells.

[36] Johnson VA, Singh EK, Nazarova LA, Alexander LD, McAlpine SR (2010). Macrocyclic inhibitors of hsp90. Curr Top Med Chem.

[37] Hanahan D, Weinberg RA (2000). The hallmarks of cancer. Cell.

[38] Zhang H, Burrows F (2004). Targeting multiple signal transduction pathways through inhibition of Hsp90. J Mol Med (Berl).

[39] Workman P, Burrows F, Neckers L, Rosen N (2007). Drugging the cancer chaperone HSP90: Combinatorial therapeutic exploitation of oncogene addiction and tumor stress. Ann NY Acad Sci.

[40] Pearl LH, Prodromou C, Workman P (2008). The Hsp90 molecular chaperone: An open and shut case for treatment. Biochem J.

[41] Eskew JD, Sadikot T, Morales P, Duren A, Dunwiddie I, Swink M, Zhang X, Hembruff S, Donnelly A, Rajewski RA (2011). Development and characterization of a novel C-terminal inhibitor of Hsp90 in androgen dependent and independent prostate cancer cells. BMC Cancer.

[42] Centenera MM, Fitzpatrick AK, Tilley WD, Butler LM (2013). Hsp90: Still a viable target in prostate cancer. Biochim Biophys Acta.

[43] Porter JR, Fritz CC, Depew KM (2010). Discovery and development of Hsp90 inhibitors: A promising pathway for cancer therapy. Curr Opin Chem Biol.

[44] Soga S, Akinaga S, Shiotsu Y (2013). Hsp90 inhibitors as anti-cancer agents, from basic discoveries to clinical development. Curr Pharm Des.

[45] Price JT, Quinn JM, Sims NA, Vieusseux J, Waldeck K, Docherty SE, Myers D, Nakamura A, Waltham MC, Gillespie MT, Thompson EW (2005). The heat shock protein 90 inhibitor, 17-allylamino-17-demethoxygeldanamycin, enhances osteoclast formation and potentiates bone metastasis of a human breast cancer cell line. Cancer Res.

